# Tunable Photonic Paints via Block Copolymer Self-Assembly and Refractive Index Engineering

**DOI:** 10.1021/acsapm.5c01430

**Published:** 2025-06-18

**Authors:** Simone Bertucci, Remi Schobinger, Liviana Mummolo, Niklas Schwarz, Paola Lova, Christoph Weder, Davide Comoretto, Ullrich Steiner, Francesco Di Stasio, Andrea Dodero

**Affiliations:** † Photonic Nanomaterials, 121451 Istituto Italiano di Tecnologia, Via Morego 30, Genoa 16163, Italy; ‡ Department of Chemistry and Industrial Chemistry, University of Genoa, Via Dodecaneso 31, Genoa 16146, Italy; § Adolphe Merkle Institute, 311305 University of Fribourg, Chemin des Verdiers 4, Fribourg 1700, Switzerland; ∥ National Center of Competence in Research Bio-Inspired Materials, Chemin des Verdiers 4, Fribourg 1700, Switzerland

**Keywords:** photonic paints, block copolymers, structural color, refractive index contrast, self-assembly

## Abstract

Structurally colored paints based on photonic microparticles offer an intriguing alternative to conventional pigment-based colorants but face persistent challenges related to limited color vibrancy and excessive light scattering. In this work, we present a scalable strategy to fabricate water-based photonic paints using poly­(2-vinylpyridine)-*block*-poly­(methyl methacrylate) (P2VP-*b*-PMMA) microparticles formed via confined self-assembly in emulsion droplets. These microparticles exhibit a concentric lamellar morphology, resulting in a photonic bandgap in the visible spectral region. Selective incorporation of 2,4,6-triiodophenol (TIPh) into the P2VP domains increases the refractive index contrast between microphase-separated domains, giving rise to tunable and more intense structural colors compared to additive-free microparticles. Detailed optical and structural characterization reveals a correlation among additive content, photonic bandgap position, and structural ordering. To reduce diffuse scattering and enhance color saturation, the broadband absorber Sudan Black B (C_29_H_24_N_6_) is then coassembled within the microparticles. These photonic pigments are finally combined with a waterborne polyurethane binder to produce robust, vividly colored coatings. The binder improves film homogeneity, reduces interparticle scattering, and enhances mechanical integrity. This multicomponent approach—integrating refractive index engineering, pigment dispersion control, and optical absorption—enables the fabrication of structurally colored paints with improved vibrancy, uniformity, and tunability.

## Graphical abstract

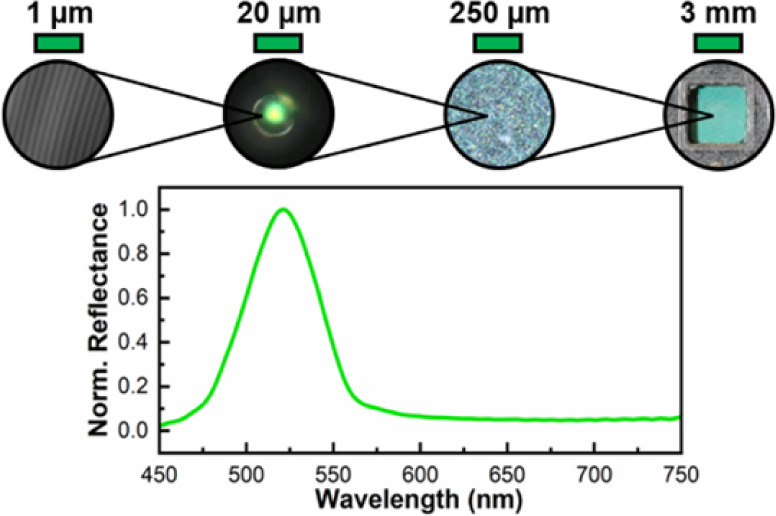

## Introduction

Colors and paints have been integral to human culture and technology since ancient times. Early paints were derived from natural substances such as plant extracts, minerals, and animal-derived pigments. In contrast, modern colorants typically rely on synthetic pigments or dyes encapsulated in a polymeric binder, allowing for improved color vibrancy and tunability.^1,2^ These colorants work by absorbing specific wavelengths of light and can be divided into two broad groups, namely inorganic and organic. Inorganic pigments are cost-effective and exhibit excellent lightfastness, but often lack high color intensity and tunability and raise toxicity concerns.^3^ Organic pigments, on the other hand, provide superior vibrancy and color purity but tend to be expensive and prone to degradation over time, resulting in color fading and bleaching. Fading requires frequent reapplication, especially for outdoor applications, and poses significant challenges in fields such as art conservation.^4^ To address these limitations, researchers have sought materials that provide bright coloration and are capable of preventing chemical or photobleaching.

Nature serves as a compelling source of inspiration, having evolved a wide variety of structural colors.^5,6^ Examples include the iridescent feathers of birds,^7^ the shimmering wings of butterflies,^8^ and the dynamic camouflage of chameleons^9^ and cephalopods.^10^ Unlike absorption-based colors, these striking optical effects result from light interactions with periodically organized nanostructures.^11^ Such structures manipulate light through diffraction, refraction, reflection, and scattering, producing more vivid and durable colors than those achieved with absorption-based colorants. Among these bioinspired approaches, structural coloration based on photonic crystals (PhCs) has attracted the most interest.^12^ Photonic crystals consist of periodic arrays of at least two dielectric materials with different refractive indices, with structural periodicities comparable to the wavelength of visible light. This periodic refractive index modulation results in the formation of photonic band gaps (PBGs), where light propagation is prevented within a specific spectral range, leading to intense selective reflection and the appearance of vivid colors. Due to these unique optical properties, PhCs have applications in diverse fields, including sensing, display technologies, passive thermal control, and anticounterfeiting.^13^ However, their direct implementation in photonic paints remains challenging.^14,15^ Previous efforts have primarily focused on distributed Bragg reflectors (DBRs) and colloidal crystals. DBRs—multilayer stacks of alternating high and low refractive index materials—allow for precise color control by modulating layer thickness.^16^ However, their angular dependence (i.e., color shifts with viewing angle) and rigid film-like nature limit their practicality for paint formulations. Meanwhile, colloidal crystals—assembled from organic or inorganic nanoparticles into ordered spherical architectures—offer scalable production and easy dispersion in polymeric matrices.^17^ Nevertheless, their limited color tunability and difficulty in achieving red hues, heterogeneity, low reflectivity, and diffuse scattering effects hinder their performance in high-quality photonic paints.

Block copolymer photonic microparticles fabricated by three-dimensional confined self-assembly within emulsion droplets have recently emerged as a promising alternative.^18–24^ Block copolymers (BCPs) consist of macromolecules containing at least two covalently bonded, chemically distinct polymer segments that undergo microphase separation to form periodic nanostructures such as lamellae, cylinders, or gyroids.^25–27^ Unlike conventional polymer blends, where phase separation occurs at the macroscale, BCPs self-assemble into well-ordered nanostructures due to the constraints imposed by the inherent molecular immiscibility of the two blocks and their covalent linkage. When confined within emulsion droplets, interfacial interactions, curvature effects, and confinement-induced morphology shifts can result in structurally colored microparticles, typically exhibiting concentric lamellar architectures.^28–32^ When dispersed in aqueous media, these colloidal PhCs can generate intense, tunable structural colors. Notably, due to their unique spherical symmetry and similarly to what is often observed for disordered colloidal crystals,^33–35^ these microparticles exhibit largely angular-independent structural coloration under diffuse illumination, which is the relevant lighting condition for most practical applications. Nonetheless, their incorporation into polymer-based photonic paints—consisting of a robust photonic pigment and a polymeric film-forming component—remains largely unexplored, mainly due to fundamental limitations that affect their optical performance. One of the main challenges is the inherently low refractive index contrast between the polymeric domains. The intensity and brilliance of structural colors depend on the refractive index difference (Δ*n*) between adjacent phases, yet most block copolymers consist of distinct segments with similar refractive indices.^36^ This weak dielectric contrast results in muted colors rather than the vibrant hues observed in high refractive index materials, such as inorganic photonic structures. In addition, uncontrolled light scattering compromises their visual impact when these microparticles are packaged in a polymeric binder. Refractive index mismatches between the particles and the surrounding medium, combined with surface roughness and particle aggregation, contribute to excessive diffuse scattering. Such an effect creates a whitish haze that dilutes the vibrancy of the structural colors, reducing their purity and brilliance.^37,38^

In this work, we present a scalable strategy for fabricating water-based photonic paints that combine BCP photonic microparticles with a high refractive index additive and a broadband absorber (Figure 1). To increase the low optical contrast of typical block copolymers, we developed a system comprising poly­(2-vinylpyridine)-*b*-poly­(methylmetacrylate) (P2VP-PMMA) and 2,4,6-triiodophenol (TIPh), where TIPh increases the Δ*n* between the P2VP and PMMA domains and tunes the structural order and associated color of the microparticles. Aqueous suspensions of the photonic pigments are combined with a commercially available waterborne polyurethane binder to develop structurally colored paints that form durable, vividly colored films upon drying. In addition, we demonstrate that the incorporation of (2,2-dimethyl-1,3-dihydroperimidin-6-yl)-(4-phenylazo-1-naphthyl)­diazene, a broadband absorbing dye commonly known as Sudan Black B (SB), into the BCP and additive formulations before the emulsification process allows for the effective suppression of unwanted scattering in the dried paints, increasing their color saturation and improving their overall optical appearance.

**Figure 1:**
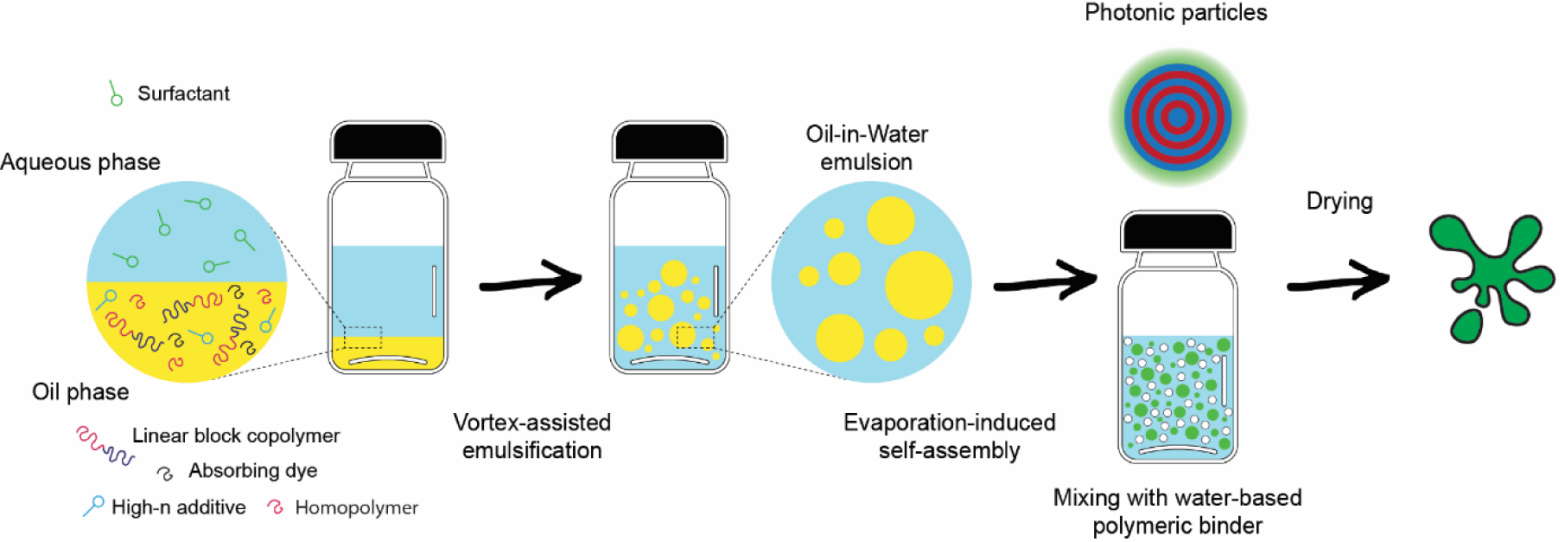
Schematic of the photonic paint fabrication process. A mixture of the BCP, a high refractive index additive, a homopolymer swelling agent, Sudan Black as a broadband absorber, and a hydrophobic solvent (oil phase) is emulsified with poly­(vinyl alcohol)-containing water (continuous phase) to form an oil-in-water (O/W) emulsion. The slow diffusion of the hydrophobic solvent through the aqueous phase causes the block copolymer chains to organize into well-defined nanostructures, eventually resulting in a suspension of solid microparticles that exhibit a vivid structural coloration. The suspension is then mixed with a water-based polyurethane binder to produce stable photonic paints that, when dried, yield bright, structurally colored films.

## Results and Discussion

Photonic microparticles with a concentric lamellar structure were prepared as previously described.^23,24^ Briefly, a chloroform solution (oil phase) of symmetric, linear P2VP-PMMA, homopolymer poly­(methyl methacrylate) (hPMMA), which is added to swell the PMMA phase, and TIPh are emulsified with an aqueous solution containing poly­(vinyl alcohol) (PVA) as a surfactant (continuous phase). The resulting oil-in-water (O/W) emulsion allows slow diffusion of the organic solvent from the oil droplets. During this process, the BCP chains undergo spherical confinement, allowing their self-assembly into concentric lamellae due to the similar block volume and the preferential interactions between the P2VP domains and the PVA surfactant.^29^ Due to the refractive index (*n*) mismatch between the different domains, the resulting microparticles exhibit a distinct photonic reflection peak located at a wavelength (λ_max_) that depends on the lamellar thickness (*D* = *d*_H_ + *d*_L_) and the refractive indices according to the Bragg–Snell law,^16^

$$\lambda_{\max}=2D\sqrt{\frac{d_{H}n_{H}^{2}}{D}+\frac{d_{L}n_{L}^{2}}{D}}$$

where H and L refer to the high and low refractive index materials, respectively. In previous work, we have shown that the simplest strategy to tune the PBG spectral position is based on the addition of amphiphilic molecules capable of selectively binding to one of the copolymer blocks via hydrogen (e.g., 3-pentadecylphenol) or ionic (e.g., 4-dodecylbenzenesulfonic acid) bonding, thereby increasing the associated volume fraction.^23^ To prevent changes in the self-assembled structure due to such variation, we also incorporated a counterbalancing amount of homopolymer with two functions.^39^ On the one hand, it maintains the symmetric self-assembled structure of the BCP (i.e., *f*_BlockA_ = *f*_BlockB_, where *f* is the volume fraction), ensuring the formation of a lamellar phase. On the other hand, it increases the mobility of the long BCP chains to produce defect-free microphase morphologies.^40^ While this approach yielded colored microparticles, the low Δ*n* limited the reflectivity, i.e., the brightness of the structural color.

To address this issue, we now employ two complementary strategies. First, we choose P2VP-PMMA as a block copolymer because it has a much higher Δ*n* between the two domains (0.13) than other commercially available BCPs (e.g., poly­(styrene)-*b*-poly­(2-vinylpyridine), Δ*n* = 0.03; poly­(styrene)-*b*-poly­(methyl methacrylate), Δ*n* = 0.1).^41^ Second, we chose 2,4,6-triiodophenol to swell the P2VP and hPMMA to swell the PMMA domains. Since this phenolic compound has a refractive index of 1.82,^42^ its selective incorporation into the high refractive index P2VP lamellae due to hydrogen bonding between the hydroxyl groups and the pyridine moieties significantly increases Δ*n*.

To study the effect of the incorporation of the additives into the block copolymer domains, differential scanning calorimetry (DSC) experiments were performed on P2VP­(TIPh)-PMMA­(hPMMA)*_x_* formulations after their complete drying, where *x* corresponds to the ratio between the number of TIPh molecules and pyridine repeat units, and hPMMA is added so that the volume of the additives is equal. The DSC curves are shown in Figure 2a. The neat block copolymer exhibits two distinct glass transition temperatures (*T*_g_) of *T*_g_^P2VP^ ∼ 97 °C and *T*_g_^PMMA^ ∼ 125 °C. These show a significant decrease upon the addition of TIPh and hPMMA as shown in Figure 2b. As predicted by the Fox equation for plasticizing a glassy polymer with a low molecular weight plasticizer,^43^ *T*_g_ decreases linearly up to *x* ∼ 0.6 (*T*_g_^P2VP^ ∼ 74 °C and *T*_g_^PMMA^ ∼ 95 °C), indicating that in this compositional regime TIPh and hPMMA are fully miscible with the P2VP and PMMA blocks, respectively.^44,45^ For higher *x*-ratios, the slope of the *T*_g_ vs *x* plot changes, suggesting that the solubility limit has been reached.

**Figure 2:**
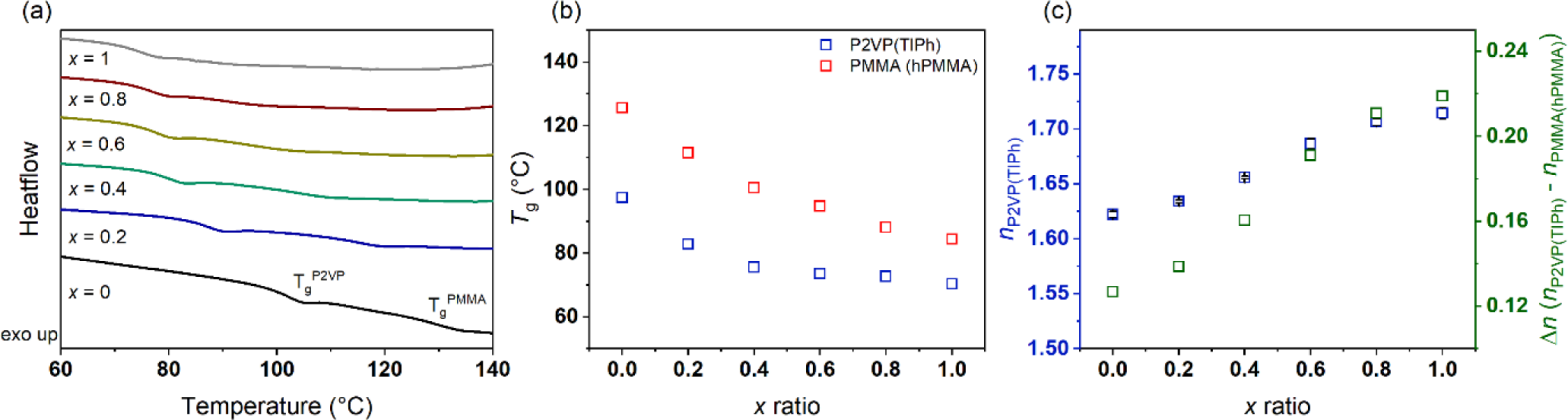
(a) DSC traces (first heating scans) of dried P2VP­(TIPh)-PMMA­(hPMMA) formulations. A clear decrease in the glass transition temperature of the P2VP and PMMA domains is observed with the addition of TIPh and hPMMA. (b) Variation of the glass transition temperature of P2VP (blue squares) and PMMA (red squares) domains with the addition of increasing amounts of TIPh and hPMMA. The progressive decrease in *T*_g_^P2VP^ indicates the existence of specific interactions between the pyridine units and the high refractive index additive. (c) Effect of TIPh addition on the refractive index of P2VP domains (blue squares) measured by spectroscopic ellipsometry and on the refractive index contrast between blocks (red squares). An almost linear increase in *n*_P2VP_ is observed with increasing TIPh content, allowing a significant increase of Δ*n* up to 0.22 when *x* = 1.

We then performed spectroscopic ellipsometry to evaluate the effective refractive index changes achieved by introducing TIPh. These experiments were performed with the hP2VP homopolymer mixed with progressive amounts of additive. Thin films were prepared by spin coating, and the ellipsometry data were fitted with the Cauchy model for transparent films (Figure S1).^46^ The refractive indices thus determined (λ = 632 nm), denoted *n*_P2VP(TIPh)_, are plotted as a function of *x* in Figure 2c, along with the Δ*n* values (Δ*n* = *n*_P2VP(TIPh)_ – *n*_PMMA(hPMMA)_). As expected, significant changes in *n*_P2VP(TIPh)_ and Δ*n* are observed, with the latter increasing from 0.13 (*n*_P2VP(TIPh)_ *^x^* ^=0^ = 1.62) for the neat BCP to 0.22 for *x* = 1 (*n*_P2VP(TIPh)_ *^x^* ^=1^ = 1.71). Similar to what was observed by DSC, at high *x*-ratios the increment effect appears to be less effective, further suggesting that for these compositional regimes some of the TIPh may phase separate from the BCP microphase morphology. Nevertheless, these results clearly confirm that the strategy employed here is a straightforward approach to tune the refractive index of block copolymers, paving the way for the fabrication of better-performing self-assembled optical nanostructures.

Based on these observations, P2VP­(TIPh)-PMMA­(hPMMA) microparticles with varying amounts of TIPh and hPMMA (expressed by the *x-*ratio) were prepared. Optical micrographs of individual particles are shown in Figure 3a. While the particles produced from the neat BCP are colorless, adding TIPh and hPMMA leads to an intense coloration in the center of the particles. In particular, the color shifts to blue (*x* = 0.2), green (*x* = 0.4), yellow (*x* = 0.6), and red (*x* = 0.8) with increasing amounts of additives. A progressive decrease in microparticle diameter is observed at higher additive loadings, which is attributed to the amphiphilic nature of TIPh. Acting similarly to a surfactant, TIPh reduces interfacial tension during emulsification, promoting the formation of smaller droplets and, consequently, smaller particles. Quantitative insight into the optical properties of the particles was obtained by microspectroscopy. The normalized reflectance spectra shown in Figure 3b confirm the existence of a well-defined photonic reflection peak for all samples (Figure S2 shows the absolute reflectance spectra for microparticles with different *x*-ratios, and Figure S3 shows the absolute reflectance spectra for several microparticles with *x* = 0.6). The linear shift toward longer wavelengths upon addition of the swelling agents is summarized in Figure 3c and is in agreement with the microscopy images (for neat BCP particles, λ_max_ ∼ 310 was calculated from the Bragg–Snell law,^16^ knowing the domain periodicity retrieved from scattering and morphological experiments and the refractive indices retrieved from ellipsometry measurements). Furthermore, a progressive broadening of the reflection peak is observed with increasing *x*-ratio. While the redshift can be attributed to thicker polymer domains, the broader peaks at high *x*-ratios can be explained by a combination of increased *Δn* and reduced of structural order, likely caused by partial phase separation of the additives from the BCP domains. To support this interpretation, finite-difference time-domain (FDTD) simulations were performed using an idealized 2D model of perfectly ordered concentric lamellae (Figure S4a). The comparison between the simulated and experimental reflectance spectra (Figure S4b) shows that the spectral position of the photonic bandgap matches well with the experimental data. Additionally, the simulated spectra significantly narrower, suggesting that the broadening observed at high *x*-ratios arises not only from the increased refractive index contrast but also from deviations from the ideal lamellar structural order.

**Figure 3:**
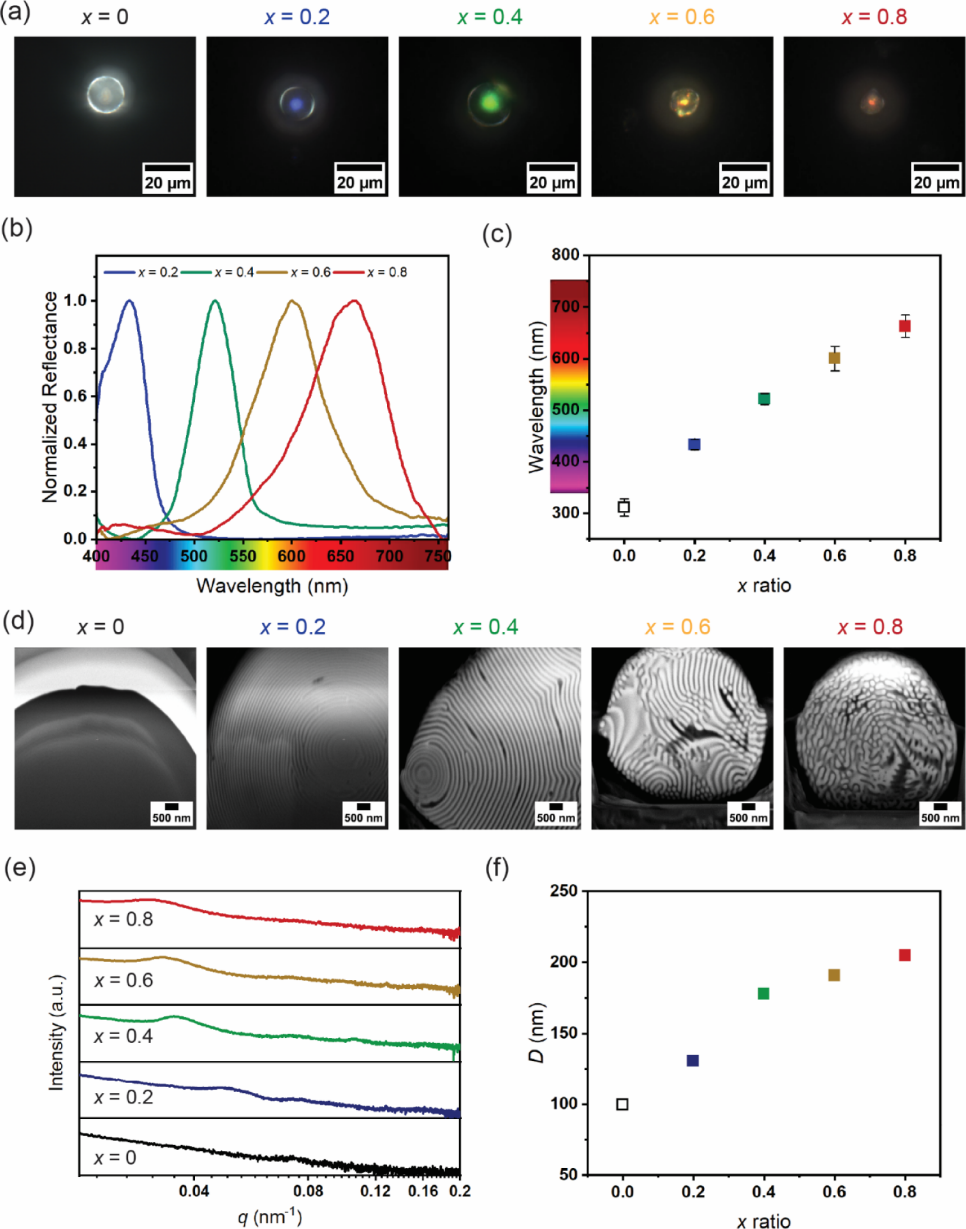
(a) Light microscopy images of photonic microparticles with increasing *x*-ratio (from left to right). While neat BCP particles are transparent (reflection peak is in the UV), the observed structural coloration at higher *x*-ratios spans the entire visible spectrum due to a progressive increase in domain periodicity and refractive index difference. (b) Normalized reflectance spectra of photonic microparticles with varying *x*-ratio. (c) Maxima of the spectra in (b) as a function of *x*-ratio. For the neat BCP particles, λ_max_ was estimated from the Bragg-Snell law. (d) FIB-SEM cross sections showing the structural arrangement of P2VP­(TIPh) (light) and PMMA­(hPMMA) (dark) domains. A transition from well-ordered concentric lamellar particles to a short-range ordered self-assembled structure is observed with increasing *x*-ratio. (e) USAXS spectra of the photonic microparticles with varying *x*-ratio obtained using synchrotron radiation. (f) Variation of domain periodicity as a function of *x*-ratio, with a progressive increase in *D* observed with increasing *x*-ratio.

To better investigate the interplay between refractive index contrast and lamellae ordering, structural characterization of the microparticles was performed using focused ion beam scanning electron microscopy (FIB-SEM) and ultrasmall angle X-ray scattering (USAXS) experiments. FIB-SEM cross sections of the particles are shown in Figure 3d, while low and high-magnification SEM top views are shown in Figure S5. Although neat P2VP-PMMA particles exhibit a clear spherical shape, the inner concentric lamellar structure is not visible due to the poor electron contrast between the block copolymer domains. Fortunately, the interblock contrast increases with increasing *x* and the BCP domains are clearly visible, further confirming the preferential incorporation of TIPh into the P2VP domains. Interestingly, at the lowest additive concentration (*x* = 0.2) the concentric lamellar structure is preserved, but at higher *x*-ratios a transformation of the internal morphology of the particles can be observed. While at intermediate additive concentrations (i.e., *x* = 0.4 and *x* = 0.6) only a change in lamellar orientation is observed, at high loadings (i.e., *x* = 0.8) the self-assembled structure shifts to a different, not readily discernible arrangement that is consistent with the formation of separate TIPh and hPMMA phases. This observation is also reflected in the top-view micrographs of the particles (Figure S5), where distinct shape variations (i.e., conical, rod-shaped, deformed) are observed. These results are due to the thermodynamics of the evaporation-induced assembly process used.^47–50^ Since the formation of solid domains starts at the oil–water interface, the establishment of strong preferential interactions between one of the blocks and the stabilizing surfactant is a mandatory requirement to ensure the growth of homogeneous, concentric lamellae. This is the case for the neat P2VP-PMMA, where the polar 2VP moieties are attracted to the PVA chains (i.e., surfactant) located at the droplet interface due to their greater hydrophilicity. On the contrary, the addition of TIPh is expected to screen such preferential interactions due to its ability to bind to the pyridine nitrogen atom, thereby changing the surface energy of the droplet and leading to significant structural changes. However, it is worth mentioning that these morphological changes are less pronounced in larger particles, suggesting that the degree of confinement also influences the internal structural evolution.

Although this complex behavior is not yet fully understood and further studies are required, the USAXS spectra (Figure 3e) indicate the presence of a certain degree of order consistent with the observed structural coloration. Indeed, a first-order scattering peak is detectable for all samples, allowing the retrieval of the corresponding domain sizes displayed in Figure 3e.^51^ Despite the fact that a marked increase in domain periodicity is observed for higher amounts of added additive (Figure 3f), again, the fact that two different linear trends are observed suggests the presence of an additive concentration threshold. Below such a value, BCP chains tend to organize into ordered lamellar structures (either concentric or stacked). On the contrary, when a critical additive concentration is reached, the structural order decreases due to the phase separation of the additives from the BCP microphase morphology, as indicated by the apparent broadening of the scattering peaks.

After their complete optical and structural characterization, the optical behavior of the green microparticles (i.e., P2VP­(TIPh)-PMMA­(hPMMA)_0.4_) in suspension was investigated. First, the effect of their concentration was evaluated visually under diffuse white illumination (Figure 4a). Concentrated microparticle suspensions show a much more intense coloration despite an increasingly whitish appearance. Reflectance (Figure 4b) and transmittance (Figure 4c) measurements were then performed to better quantify the color intensity. The reflectance spectra show a well-defined reflectance peak at λ ∼ 540 nm for all samples, the intensity of which decreases as the microparticle concentration decreases. As expected, this is due to the decrease in density of the photonic microspheres, leading to weaker reflection and increased light transmission. Similarly, the diffuse scattering, which can be evaluated from the intensity of the spectral background, shows a clear dependence on the microparticle concentration. Closer examination of the transmission spectra reveals additional details about the light-matter interactions in these suspensions. At low concentrations, high transmittance is observed throughout the visible range (up to nearly 100% for the 0.01 wt % suspension), indicating minimal absorption and the predominance of weak Mie scattering effects due to the relatively large particle size, resulting in directional reflection.^52^ However, as the particle concentration increases, there is a transition from single to multiple scattering regimes with increased particle interactions leading to incoherent multiple scattering, explaining the increased whiteness and reduced structural color purity.^53^ In addition, at high particle concentrations, the transmission spectra exhibit an extended tail in the lower wavelength region, most likely caused by increased short wavelength scattering due to a combination of Rayleigh and Mie scattering effects.^54^ Similar experiments were performed for the other compositions (i.e., *x*-ratios of 0.2, 0.6, and 0.8), with the data showing similar behavior (Figure S6).

**Figure 4:**
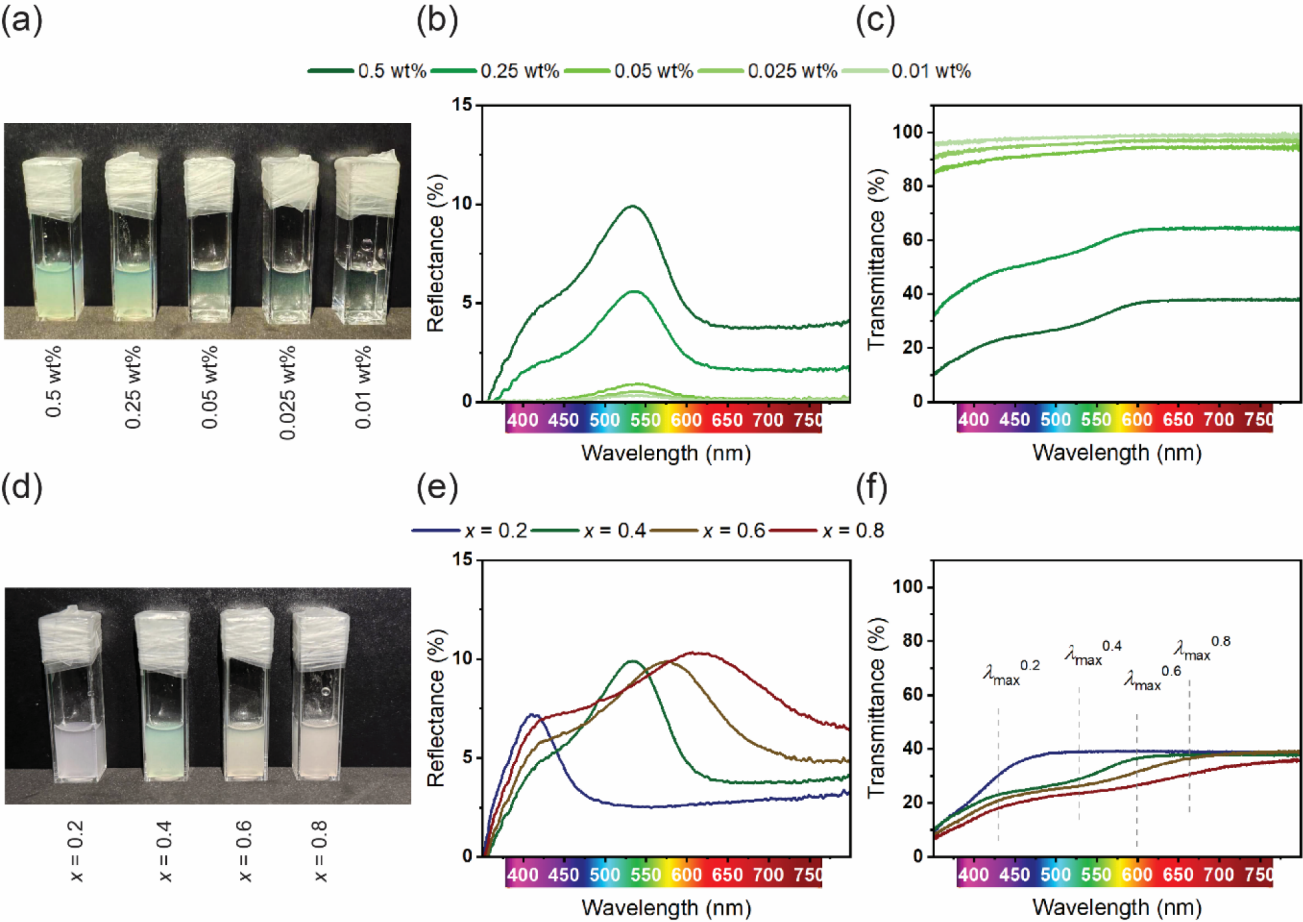
(a) Image of green microparticle suspensions (*x* = 0.4) under diffuse illumination showing the effect of particle concentration on color intensity (concentration decreases from left to right) with only a faint green hue visible at the lowest concentration. (b) Reflectance and (c) transmittance spectra of green microparticle suspensions (*x* = 0.4), where the maxima and minima correspond to the photon band gap, respectively. Higher particle concentration leads to much more pronounced scattering, corresponding to a whitish appearance. (d) Image of concentrated particle suspensions (*x* = 0.2, 0.4, 0.6, and 0.8) under diffuse illumination showing different colors. (e) Reflectance and (f) transmittance spectra of concentrated microparticle suspensions showing the variation of the reflectance peak maximum across the visible spectrum.

A comparison of highly concentrated suspensions of particles with different *x*-ratios is shown in Figure 4d, demonstrating that their colors span the entire visible spectrum. This is also evident from the corresponding reflectance (Figure 4e) and transmittance (Figure 4f) spectra. While a clear redshift of the reflection maxima and transmission minima is observed with increasing *x*-ratio, the scattering phenomena also seem to be influenced by the particles under analysis. With increasing *x*-ratio, the incorporation of higher TIPh and hPMMA content alters the internal nanostructure, effectively increasing the effective refractive index and leading to a redshift of the reflection peak. However, the higher the amount of additives, the greater the structural and shape deformations that occur during the self-assembly process. Indeed, as shown by FIB-SEM and USAXS measurements (Figure 3d,e), the self-assembled structure loses its long-range order and the particles adopt an irregular shape. Both aspects are expected to promote light scattering instead of reflection, thus negatively affecting the overall optical properties of the suspensions. Such structural irregularities disrupt the periodicity required for efficient Bragg reflection, leading to increased incoherent scattering and contributing to the observed white appearance.^55^

With the goal of developing the microsphere dispersion into a photonic paint, a polymeric binder was introduced into the system. The binder provides mechanical integrity to the paint, ensuring adhesion to substrates, increasing environmental durability, and preventing scratching.^64^ In addition, it plays a critical role in determining the optical response by influencing the refractive index contrast between the microparticles and their surrounding medium.^56^ This contrast directly affects key optical properties such as reflectance and color saturation of the structural coloration. Finally, the binder affects the spatial arrangement of the photonic particles by influencing their packing density and degree of order during film formation. Unlike dispersions in water, where Brownian motion and electrostatic interactions govern the particle distribution, the polymer matrix introduces additional constraints such as viscosity effects and interactions at the particle-binder interface. These factors can result in differences in the final optical appearance of the paint compared to its aqueous precursor and should be considered.^57^

Here, we investigated the use of a commercially available water-based polyurethane (WBPU) binder, which offers several attractive features. Because the binder is water-based, no additional solvents are required, eliminating potential particle swelling and associated changes in the intrinsic optical properties of the photonic microparticles.^19,20^ In addition, the elimination of organic solvents significantly reduces the environmental impact of the formulation, making the paint suitable for broader applications.^58^ Figure 5a shows images of films prepared by drop casting equal volumes of a particle suspension and a particle/binder formulation with the same microparticle concentration (i.e., particle concentration 1 wt %, binder concentration 15 wt %). While homogeneous coloration is obtained in both cases, the presence of the binder significantly increases the saturation of the green color. The difference in appearance is due to two different effects. First, the refractive index of the WBPU (*n* ∼ 1.55–1.6)^59^ is much closer to that of the photonic microparticles than to that of air (*n* ∼ 1). This reduces the refractive index mismatch between the photonic microparticles and the surrounding dielectric environment, thereby diffusing scattering effects. Second, the binder is expected to limit the aggregation of the microparticles and minimize drying-induced stresses, thus reducing the formation of “scattering centers” at the interparticle interface that are a source of broadband light scattering.^60^ Figure 5b, which shows optical microscopy images of the dried films, confirms the effect of the binder on the final film morphology and optical properties. In the absence of the polymeric binder, structural inhomogeneities negatively affect the optical response by introducing variations in reflectance and color uniformity. Conversely, when the aqueous polyurethane binder is included, the film appears more uniform and continuous, indicating that the polymeric matrix plays a critical role in maintaining particle dispersion and spatial organization during drying.

**Figure 5:**
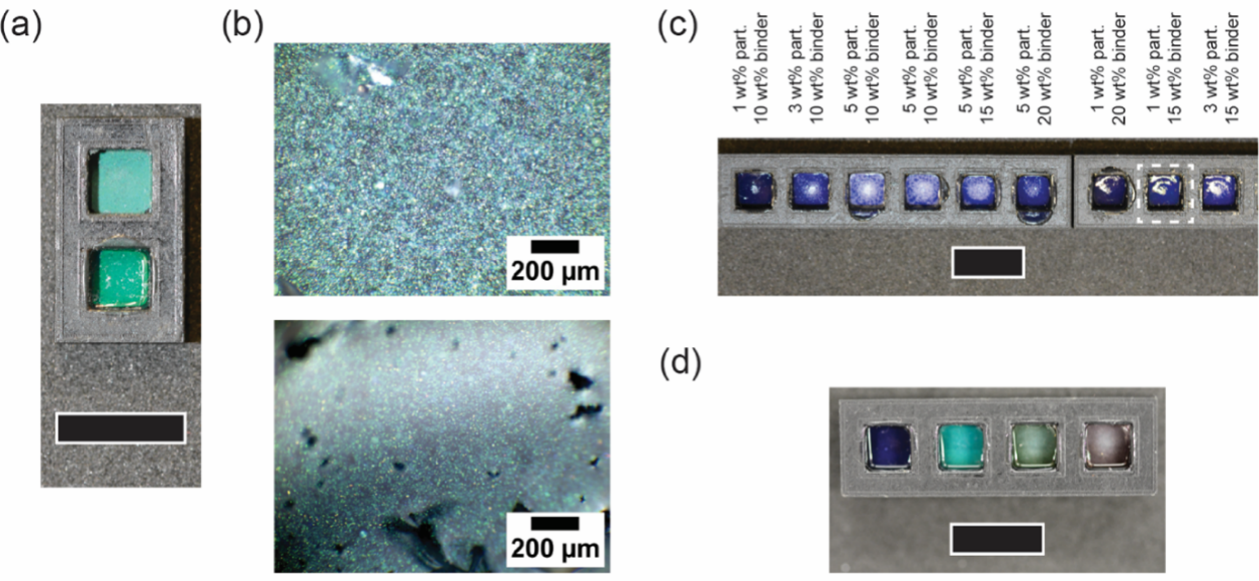
(a) Digital image under diffuse illumination and (b) light microscopy images obtained using a 20× objective of photonic films prepared by drying a green (*x* = 0.4) particle suspension without (top) and with (bottom) WBPU binder (particle concentration 1 wt %, binder concentration 15 wt %). The presence of the binder significantly improves the color brightness and overall film appearance. (c) Image of photonic films under diffuse white illumination prepared by drying blue (*x* = 0.2) particle suspensions. Particle (1–3 wt %) and binder (10–20 wt %) concentrations were varied as indicated in Table S2. (d) Image of photonic films under diffuse illumination prepared by drying blue, green, yellow, or red photonic (*x* = 0.2, 0.4, 0.6, 0.8) paints (particle concentration 1 wt %, binder concentration 15 wt %). The color intensity decreases for yellow and red films due to the lower structural order of the photonic microparticles. Unless otherwise indicated, scale bars are 10 mm.

Since these observations highlight the critical role of the binder in influencing the optical properties of the final photonic paint, we optimized the particle and binder concentrations in the paint formulation (see Table S2 for details). Figure 5c shows images of dried films prepared from paints in which the particle concentration (1–3 wt %) and the WBPU concentration (10–30 wt %) were varied. The corresponding optical microscopy images are shown in Figure S7. While the binder concentration does not significantly affect the appearance of the films, a high particle concentration results in improved color brightness, but marked inhomogeneity and a whitish appearance. Based on an empirical evaluation of these films, a particle concentration of 1 wt % and a WBPU concentration of 15 wt % are considered to provide the best optical appearance and are used for blue, green, yellow, and red colored films (Figure 5d), whose optical microscopy images are shown in Figure S8. The blue and green films show intense coloration, while the yellow and especially the red film appear less brilliant. This is consistent with the common challenges in structural colors, where red hues are particularly difficult to achieve and is mostly due to the decrease in particle structural order, which reduces the intensity of the reflected light and widens the reflection peak (Figure S2), and the increasing refractive index mismatch between the polymeric binder and the particles with increasing *x*-ratio.

Another critical factor to consider in improving the overall optical appearance of photonic paints is the reduction of diffuse scattering phenomena.^37,38,61,62^ Figure 6a shows the effect of the substrate on which the same green paint (i.e., *x* = 0.4, particle concentration 1 wt %, WBPU concentration 15 wt %) is dried. While an intense green coloration is clearly seen on a black background, only a barely detectable hue is seen when the paint is applied to a white substrate. To overcome this problem, a simple bioinspired strategy can be applied, which consists of combining structural coloration and broadband absorption. Indeed, some of the most brilliant colors in nature arise from photonic structures that contain absorbing pigments that maximize contrast without significantly affecting the color intensity.^63^ One of the most accessible strategies is the addition of “black” nanoparticles—such as carbon black or iron nanoparticles—directly into the photonic structure, but these can very often aggregate, providing little or no color enhancement. Moreover, the presence of such nanoparticles can affect the self-assembly process of BCPs, and this renders the fabrication of photonic structures challengeing.^64–68^

**Figure 6:**
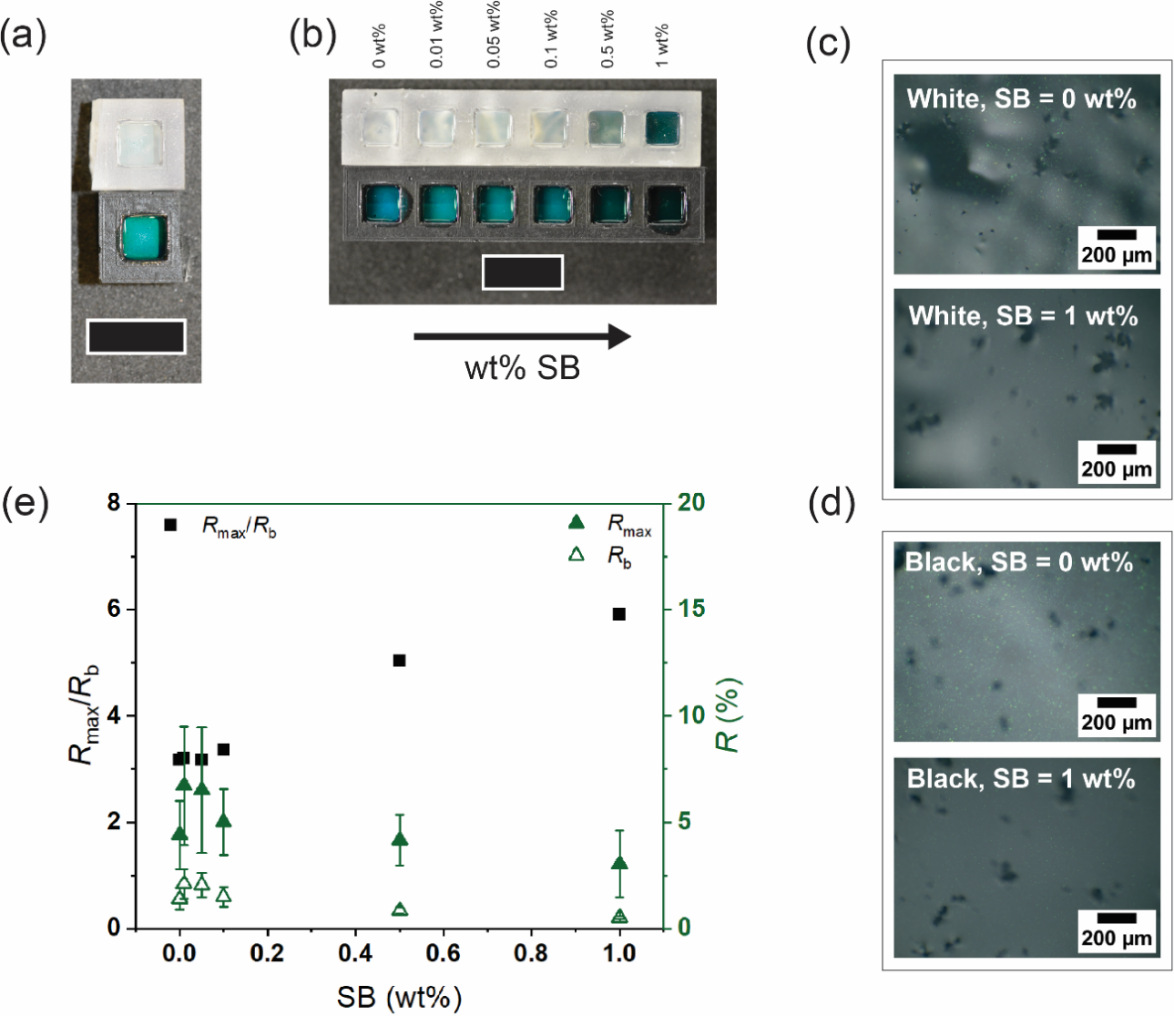
(a) Image of photonic films under diffuse white illumination obtained by drying a green photonic paint (*x* = 0.4, particle concentration 1 wt %, binder concentration 15 wt %) on a white (top) or black (bottom) substrate. While the black substrate produces an intense green color, a barely discernible hue is observed on the white substrate due to poor color contrast. (b) Image of photonic films under diffuse illumination obtained by drying an SB-loaded green photonic paint (*x* = 0.4, particle concentration 1 wt %, binder concentration 15 wt %) on a white (top) or black (bottom) substrate (SB concentration increases from left to right). A progressive improvement in color contrast is observed with increasing absorber concentration. (c,d) Light microscopy images taken with a 20× objective of photonic films prepared by drying a green photonic paint (*x* = 0.4, particle concentration 1 wt %, binder concentration 15 wt %) without absorber (top) and with the highest absorber concentration (bottom) on a (c) white or (d) black substrate. (e) Variation of peak reflectance (*R*_max_) and background intensity (*R*_b_) and their ratio (*R*_max_/*R*_b_) for single green photonic microparticles loaded with different amounts of SB. Unless otherwise indicated, scale bars are 10 mm.

As a much simpler alternative, we propose here the incorporation of small amounts of the black organic dye Sudan black B (SB) directly into the formulation used to prepare the block copolymer microparticles. The SB is expected to coassemble with the BCP and swelling agents to provide photonic microparticles with improved optical appearance. Using the same microparticle preparation process as before and selecting green microparticles (*x* = 0.4) for these experiments, SB is added at a concentration between 0.1% and 1% with respect to the total weight of the block copolymer, TIPh and hPMMA additive formulations. Optical microscopy images and reflectance spectra of individual SB-loaded particles prepared in this manner are shown in Figures S9 and S10, respectively, while images of dried photonic films on a white and black substrate are shown in Figure 6b. Although there are no major differences in the optical microscopy images of individual particles at different SB concentrations, their spectroscopic characterization reveals several interesting findings. First, at low SB content (i.e., 0.01–0.1 wt %), the particles show a higher reflectance intensity than the SB-free particles. This large, unexpected increase in reflectance (almost a factor of 2) is puzzling and requires further investigation, as it might arise from a nontrivial combination of optical contrast enhancement, scattering suppression, local resonance effects, and structural modifications. However, when the SB concentration is increased above 0.1 wt %, the reflectance intensity decreases steadily with the SB content as absorption becomes dominant. Second, the photonic reflection band becomes sharper with increasing SB content, and a slight blue shift is observed. The latter effect is consistent with the fact that SB can induce small structural changes and thus affect the optical response of the microparticles. However, the effect is limited, and the focus should be on the scattering reduction phenomenon.

This is evident when observing the photonic films obtained by drying the different photonic paints. For the films obtained on a black substrate, according to the reflectance spectra described above, increasing the absorber concentration first leads to an obvious increase in color vibrancy, followed by a progressive darkening of the displayed coloration. For the white substrates, while low SB loadings are not sufficient for the appearance of any noticeable hues, high concentrations are able to effectively suppress diffuse scattering, resulting in a bright, intense structural coloration.

The effect of the absorber is also studied by performing optical microscopy on the obtained photonic films, the resulting images are shown in Figure 6c (top panel white substrate, no SB; bottom panel white substrate, SB at highest concentration) and Figure 6d (top panel black substrate, no SB; bottom panel black substrate, SB at highest concentration). Regardless of the substrate used, a diffuse whitish coloration of the bare microparticle film is observed, which disappears upon the addition of the absorber. A better understanding of the interplay between reflection and scattering can be obtained by evaluating the reflection peak (*R*_max_) and the background intensity (*R*_b_) reflected by the spectra shown in Figure S10. While they follow the same trend as previously described (i.e., an initial increase in intensity followed by a continuous decrease), their ratio *R*_max_/*R*_b_ shows the opposite behavior with a steady increase as the SB loading becomes more pronounced. As shown in Figure 6e, while both *R*_max_ and *R*_b_ decrease with increasing absorber content, their ratio increases, reflecting the improvement in color contrast and optical appearance. These results indicate that reflection becomes the dominant phenomenon at the expense of diffuse scattering.

## Conclusions

In this study, we demonstrated a scalable approach for fabricating water-based photonic paints using block copolymer photonic microparticles embedded with high refractive index additives and broadband absorbers. By overcoming key limitations of polymer-based structural color paints, namely low refractive index contrast and excessive light scattering, we achieved a significant improvement in optical performance. First, the strategic and selective incorporation of 2,4,6-triiodophenol into P2VP blocks as a high refractive index additive significantly increases the refractive index contrast between the block copolymer domains within the fabricated microparticles. This enhancement results in a noticeable increase in color intensity and vibrancy, and our optical characterizations confirm tunable structural coloration across the visible spectrum. In addition, we demonstrate that a polymeric binder improves the uniformity and optical performance of the resulting photonic films by drying paint formulations containing the microparticles and binder. In addition, the binder significantly improves the mechanical stability of the dried films. The use of a water-based polyurethane binder not only facilitates homogeneous film formation but also mitigates particle aggregation and reduces the refractive index mismatch between the particles and their immediate environment, resulting in reduced scattering effects. Finally, to further enhance color purity and brilliance, we incorporated a black pigment as a broadband absorber to counteract unwanted diffuse scattering. The synergy between structural coloration and controlled absorption results in another remarkable improvement in color saturation, especially on white substrates where scattering-induced whitening is a persistent challenge. Our results underscore the fundamental role of selective absorption in enhancing the visual impact of photonic coatings. Our findings provide a solid foundation for next-generation photonic paints poised for transformative applications in display technologies, anticounterfeiting, and aesthetic coatings. By seamlessly integrating BCP self-assembly, refractive index engineering, and broadband absorption, we are contributing to the advancements in innovative functional coatings. In the future, our research will refine formulation strategies to improve long-term stability, investigate other high refractive index additives, explore alternative and sustainable block copolymers and polymeric binders, and optimize color vibrancy for yellow and red hues, setting the stage for even more sophisticated and performant photonic coatings.

## Materials and Methods

### Materials

Poly­(2-vinylpyridine)-*b*-poly­(methyl methacrylate) (P2VP-PMMA) with M̅_n_ = 235-*b*-220 kg/mol, poly­(methyl methacrylate) homopolymer (hPMMA) with M̅_n_ = 5000 kg/mol, and poly­(2-vinylpyridine) homopolymer (hP2VP) with M̅_n_ = 5300 kg/mol were obtained from Polymer Source. Poly­(vinyl alcohol) (PVA) with M̅_n_ = 13–23 kg/mol and a degree of hydrolysis of 87–89%, 2,4,6-triiodophenol (TIPh) 97%, and (2,2-dimethyl-1,3-dihydroperimidin-6-yl)-(4-phenylazo-1-naphthyl)­diazene (Sudan Black B (SB)) were purchased from Sigma-Aldrich. Amylene-stabilized chloroform (CHCl_3_) 99+% for spectroscopy was purchased from Fischer Scientific. Aqueous polyurethane binder (LUPHEN700) was kindly provided by BASF. Milli-Q water with a conductivity of 0.055 μS/cm was prepared in-house and used for all experiments.

### Methods

#### Preparation of Photonic Particles, Photonic Paints, and Photonic Films

P2VP-PMMA, TIPh, and hPMMA were dissolved separately in CHCl_3_ with stirring at a concentration of 10 mg/mL, and the resulting solutions were mixed at different ratios according to Table S1. The content of TIPh in the final particles is expressed as *x*-ratio, which corresponds to the molar ratio between TIPh molecules and P2VP repeat units. The content of hPMMA in the final particles was calculated so that the volume of the two additives (i.e., TIPh and hPMMA) was kept equal. PVA was solubilized in water at a concentration of 10 or 3 mg/mL by stirring the mixture at 85 °C for a few hours.

To prepare photonic microparticles, 250 μL of the BCP mixtures were transferred to a 7 mL glass vial and emulsified with 2.5 mL of the 10 mg/mL aqueous PVA solution by manually shaking the vial for 15 s. The resulting oil-in-water (O/W) emulsions were then poured into Petri dishes (5 cm diameter) containing 15 mL of the 3 mg/mL aqueous PVA solution. The Petri dishes were covered and left in a well-ventilated hood for 48 h to ensure complete evaporation of the organic solvent. The resulting particle suspensions were centrifuged (5000 rpm, 15 min), but never dried, and washed several times with water. Finally, water was added to obtain master suspensions with a particle concentration of 5 wt % (calculated from the dry weights of P2VP-PMMA, TIPh, and hPMMA used). For the SB-containing particles, the absorber was directly solubilized in the BCP mixture at a concentration of 0.01–1 wt % with respect to the solid content.

To prepare photonic paints, the master suspensions were mixed with an aqueous solution of the WBPU binder (37 wt % solid content) and additional water to obtain paints with a particle concentration of 1–3 wt % and a WBPU concentration of 10–20 wt % (Table S2). To fabricate photonic films, the above paints were drop cast onto black or white 5 × 5 mm^2^ 3D printed molds and dried at room temperature.

#### Characterization Techniques

Differential scanning calorimetry (DSC) experiments were performed on approximately 5 mg of dried samples using a Mettler Toledo DSC 2 STAR system under nitrogen flow in the temperature range of −50–150 °C at a heating rate of 10 °C/min. The first heating scans are shown.

Spectroscopic ellipsometry was performed using a VASE ellipsometer from J.A. Woollam Co. Inc. with an angle of incidence of 75° on hP2VP films mixed with TIPh. These films were prepared by spin-coating 5 mg/mL BCP solutions in chloroform using a volume of 100 μL, a spinning time of 90 s, and a spinning speed of 8000 rpm.

Light microscopy of individual microparticles was performed using a customized microscope (ZEISS Axio Scope.A1) equipped with a CCD camera (Point Gray GS3-U3-28S5C-C) calibrated against a standard white diffuser and a halogen lamp (Zeiss HAL100) as a light source. Micrographs were taken in reflection mode in a bright field configuration using a 50× objective (Zeiss LD EC Epiplan-Neofluar, NA = 0.8). Reflection spectra of individual microparticles were measured by coupling the microscope and objective to a diode array spectrometer (Ocean Optics QEPro) using an optical fiber confocally positioned at the image plane of the microscope (Avantes QP230-2-XSR, 230 μm core size). An aluminum mirror was used as a reference. Photonic microparticles were characterized in water suspensions on optical glass slides covered with a glass coverslip. Optical microscopy on photonic films was performed with the same setup using a 20× (Zeiss LD EC Epiplan-Neofluar, NA = 0.22) or 10× objective (Zeiss LD EC Epiplan-Neofluar, NA = 0.25).

Reflectance and transmittance measurements of microparticle suspensions were performed in PMMA cuvettes with an integrating sphere connected by two optical fibers (OceanOptics, QP600-2-SR-BX and OceanOptics, QP400-2-XSR, respectively) to a high power xenon light source (OceanOptics, HPX-2000) and a UV–vis spectrometer (OceanInsight, Flame-T-XR1-ES). A white diffuser (SphereOptics, Zenith Polymer SG 3051, 99%) was used as a reflectance standard.

Modeling of the photonic microparticle optical properties was based on finite-difference time-domain (FDTD) simulations using commercial Ansys Lumerical software. The rotational symmetry of the particles allowed for simulating cross sections in 2D, considering plane-wave illumination (400–800 nm), a mesh size of 10 nm, and PML boundaries. A power monitor placed behind the source was used to collect the reflected light. The upper half of photonic microparticles with a diameter of 20 μm and immersed in water was analyzed.

Structural analysis of the photonic microparticles was performed by focused ion beam scanning electron microscopy (FIB-SEM) using a Thermo Scientific Scios 2 DualBeam FIB-SEM (FEI, Eindhoven, The Netherlands). Particle suspensions were first drop cast onto aluminum stubs covered with conductive carbon tape and coated with a 4 nm gold layer. Half of the microspheres were milled with a Ga^+^ ion beam set at 30 kV acceleration voltage, and the cut surface was imaged with an in-lens T1 detector (A+B composite mode, backscattered electrons) set at 5 kV.

Ultrasmall-angle X-ray scattering (USAXS) measurements were performed on the ID02 beamline at the European Synchrotron Radiation Facility (ESRF) in Grenoble. Circular bulk samples with an average thickness of 1 mm and a diameter of 4 mm were prepared by drop casting concentrated particle suspensions into polytetrafluoroethylene (PTFE) disks sealed on both sides with Kapton tape (DuPont).

## Supplementary Material



## Data Availability

Data are available free of charge at https://doi.org/10.5281/zenodo.15669245.

## Notes

Additional figures supporting the presented data and tables reporting experimental details—Figure S1: Refractive index dispersion of polymeric thin films at increasing content of TIPh additive; Figure S2: Reflectance spectra of single BCP microparticles at different additive contents; Figure S3: Reflectance spectra of various single microparticles with the same composition; Figure S4: FDTD simulations of the optical response of a single photonic microsphere compared to experimental results for the different formulations; Figure S5: SEM micrographs of self-assembled microparticles with increasing additive content; Figure S6: Reflectance and transmittance spectra of aqueous dispersions of microparticles at different concentrations for all formulations utilized; Figure S7: Optical microscope images of blue photonic thin films for different ratios between microparticles and polymeric binder; Figure S8: Optical microscope images for all optimized formulations; Figure S9: Optical microscope images of green microparticles dried films for different contents of black absorber; Figure S10: Reflectance spectra of green microparticles dried films for varying content of black absorber; Table S1. Detailed formulations for TIPh-loaded microparticles; Table S2: Concentrations employed for the optimization of the photonic pigments (PDF)
