# Supplementary material for: Tunable Photonic Paints via Block Copolymer Self-Assembly and Refractive Index Engineering
Source: ACS Appl Polym Mater. 2025 Jun 18;7(13):8743–54. doi: 10.1021/acsapm.5c01430 (PMC12261279; doi:10.1021/acsapm.5c01430)
Supplement: Supplementary file 1 [file ap5c01430_si_001.pdf]

Supporting Information

Tunable Photonic Paints via Block-Copolymer Self-Assembly and Refractive Index Engineering

*Simone Bertucci[1,2,3] Remi Schobinger,[3] Liviana Mummolo,[3,4] Niklas Schwarz,[3,4] Paola Lova,[2] Christoph Weder,[3,4] Davide Comoretto,[2] Ullrich Steiner,[3,4] Francesco Di Stasio,[1] Andrea Dodero[2,3,4]**

[1] Photonic Nanomaterials, Istituto Italiano di Tecnologia, Via Morego 30, 16163 Genoa, Italy

[2] Department of Chemistry and Industrial Chemistry, University of Genoa, Via Dodecaneso 31, 16146, Genoa, Italy

[3] Adolphe Merkle Institute, University of Fribourg, Chemin des Verdiers 4, 1700, Fribourg, Switzerland

[4] National Center of Competence in Research Bio-Inspired Materials, Chemin des Verdiers 4, 1700, Fribourg, Switzerland

* corresponding author: Andrea Dodero

andrea.dodero@unifr.ch

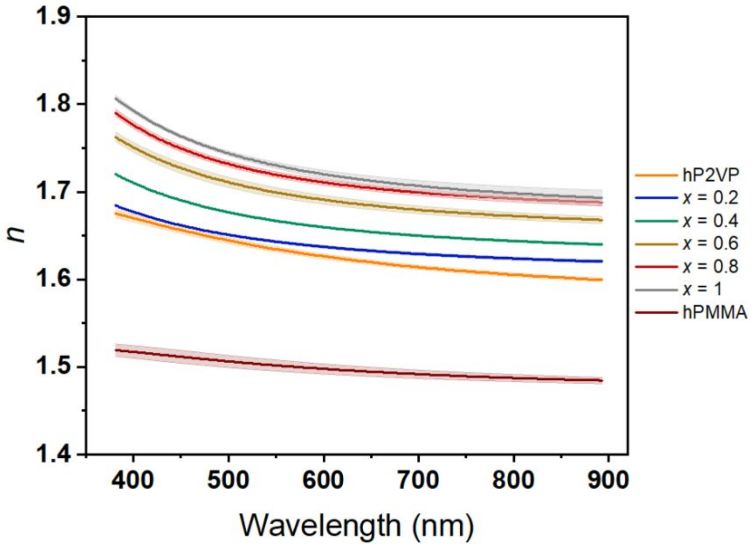

Figure S1. Refractive index dispersion for thin polymeric films on silicon substrates prepared with hP2VP(TIPh) formulations at varying x-ratios. hP2VP and hPMMA films are reported for reference. Increasing the content of TIPh in the hP2VP films leads to a considerable increase in the refractive index.

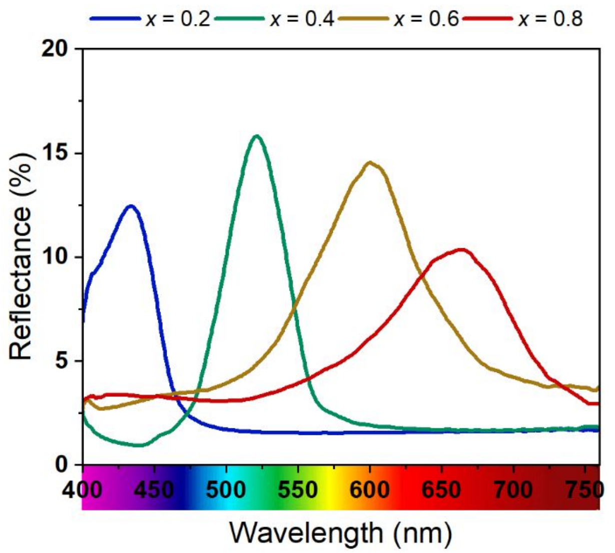

Figure S2. Reflectance spectra of single P2VP(TIPh)-PMMA(hPMMA)$_x$ particles at varying x-ratio. A higher additive concentration induces a consistent red-shift of the photonic bandgap spectral position due to the increased structural periodicity, as well as a strong broadening of the reflectance peaks because of the greater refractive index contrast between the block copolymer domains and lower ordering.

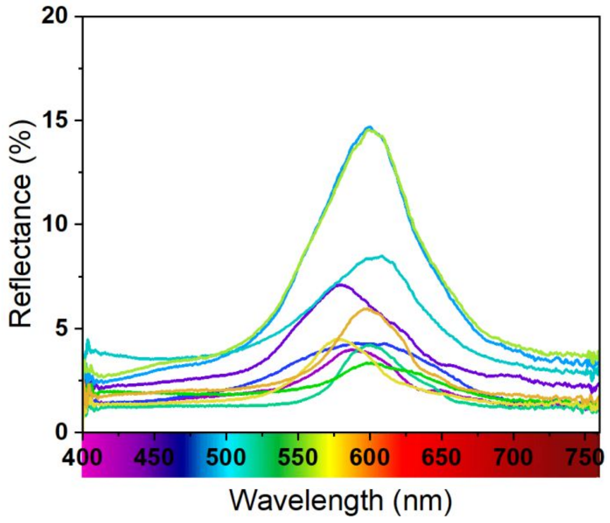

Figure S3. Reflectance spectra of several single P2VP(TIPh)-PMMA(hPMMA)$_{0.6}$ microparticles showing the reproducibility of the obtained structural coloration.

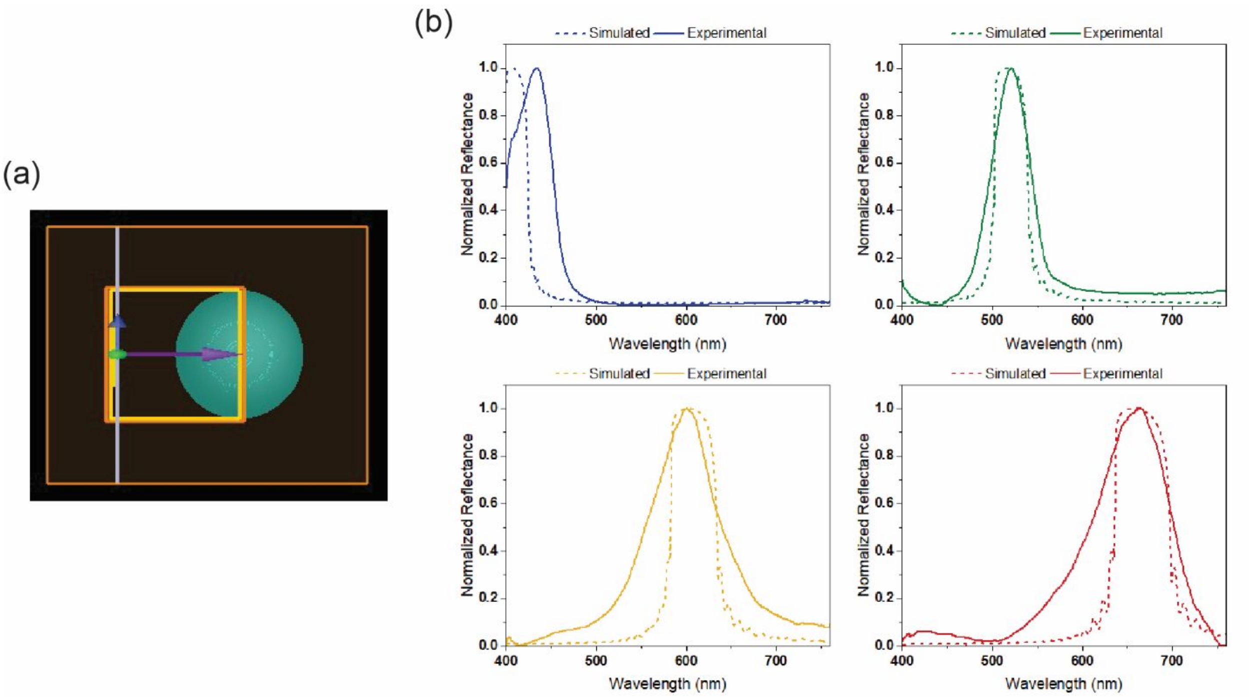

Figure S4. (a) FDTD simulation model showing a photonic model sphere, the incident light source (white), the incidence direction (purple), the polarization vector (blue), and the reflection monitor (yellow). (b) Comparison between the normalized experimental spectra (continuous lines) and normalized simulated spectra (dashed lines).

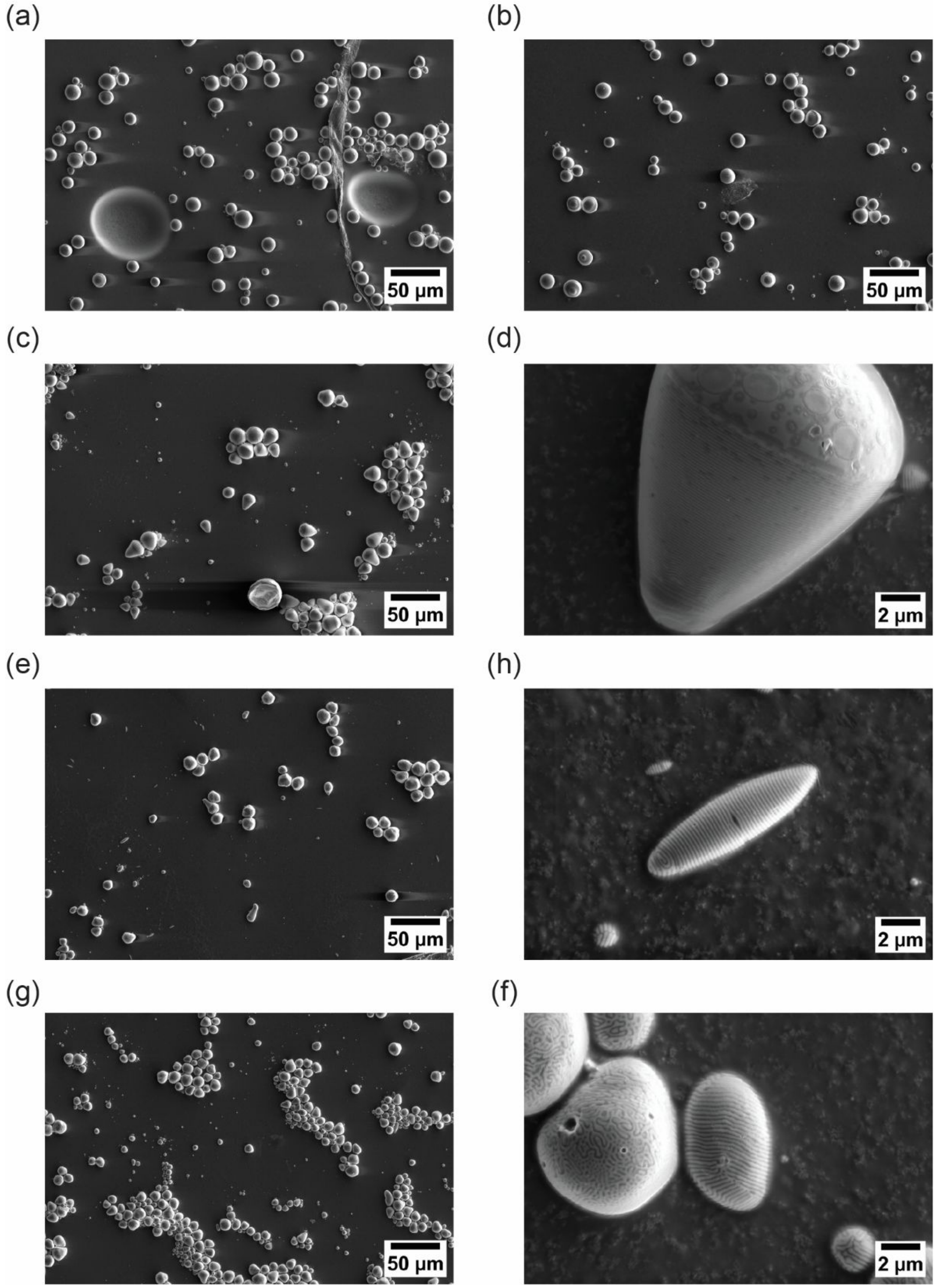

Figure S5. SEM micrographs of (a) P2VP-PMMA microparticles, (b) P2VP(TIPh)-PMMA(hPMMA)$_{0.2}$ microparticles, (c – d) P2VP(TIPh)-PMMA(hPMMA)$_{0.4}$ microparticles, (e – f) P2VP(TIPh)-PMMA(hPMMA)$_{0.6}$ microparticles, and (g – h) P2VP(TIPh)-PMMA(hPMMA)$_{0.8}$ microparticles. Spherical particles are obtained for bare P2VP-PMMA or at low additive concentration, while deformed ones are observed at high TIPh and hPMMA content. In particular, cone-like and elongated microparticles with a stacked lamellar configuration are observed due to changes of the block colymer-surfactant interations at the oil-water interface.

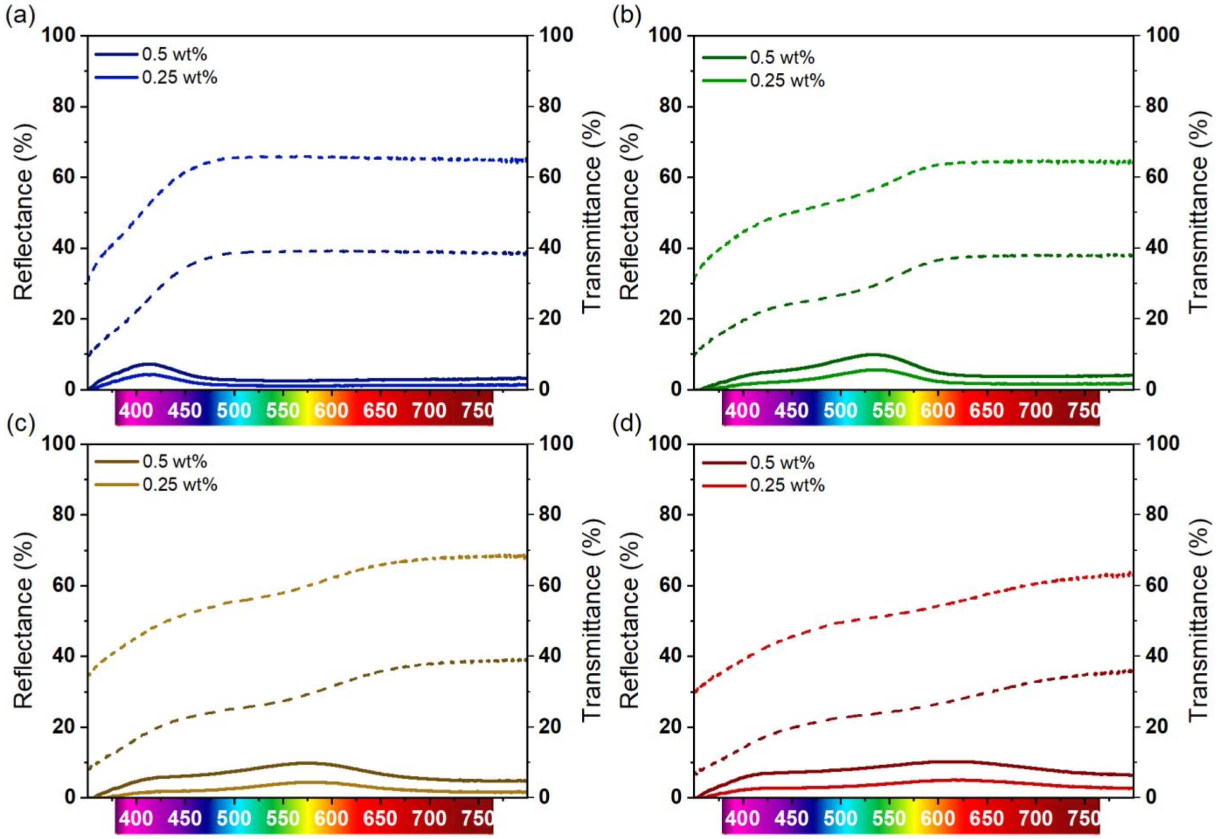

Figure S6. Reflectance (continuous lines) and transmittance (dotted lines) spectra of aqueous suspensions at varying concentrations of (a) P2VP(TIPh)-PMMA(hPMMA)$_{0.2}$ microparticles, (b) P2VP(TIPh)-PMMA(hPMMA)$_{0.4}$ microparticles, (c) P2VP(TIPh)-PMMA(hPMMA)$_{0.6}$ microparticles, and (d) P2VP(TIPh)-PMMA(hPMMA)$_{0.8}$ microparticles.

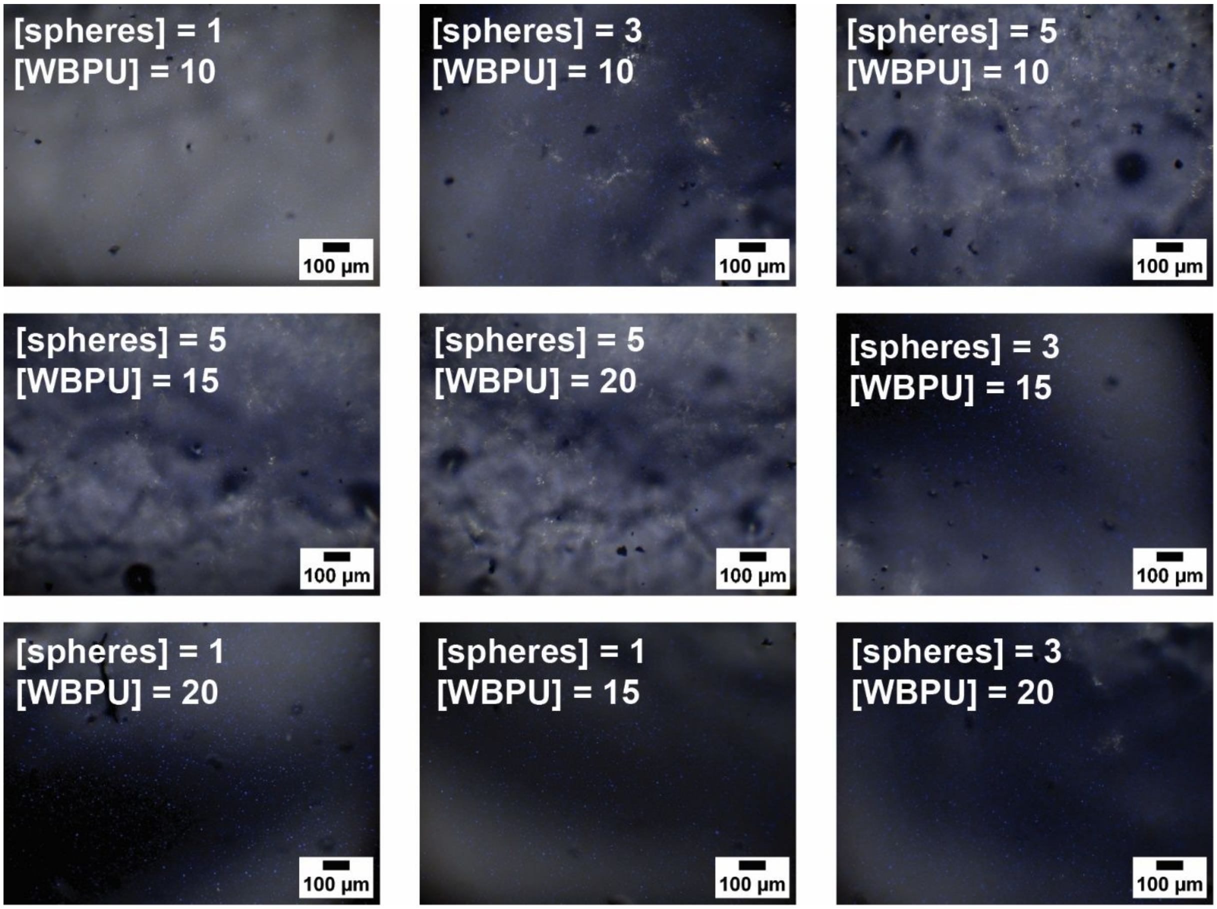

Figure S7. Optical microscopy images of blue (x = 0.2) photonic films obtained by drying aqueous formulations of microparticles and polymeric binder. Component concentrations are reported on the images.

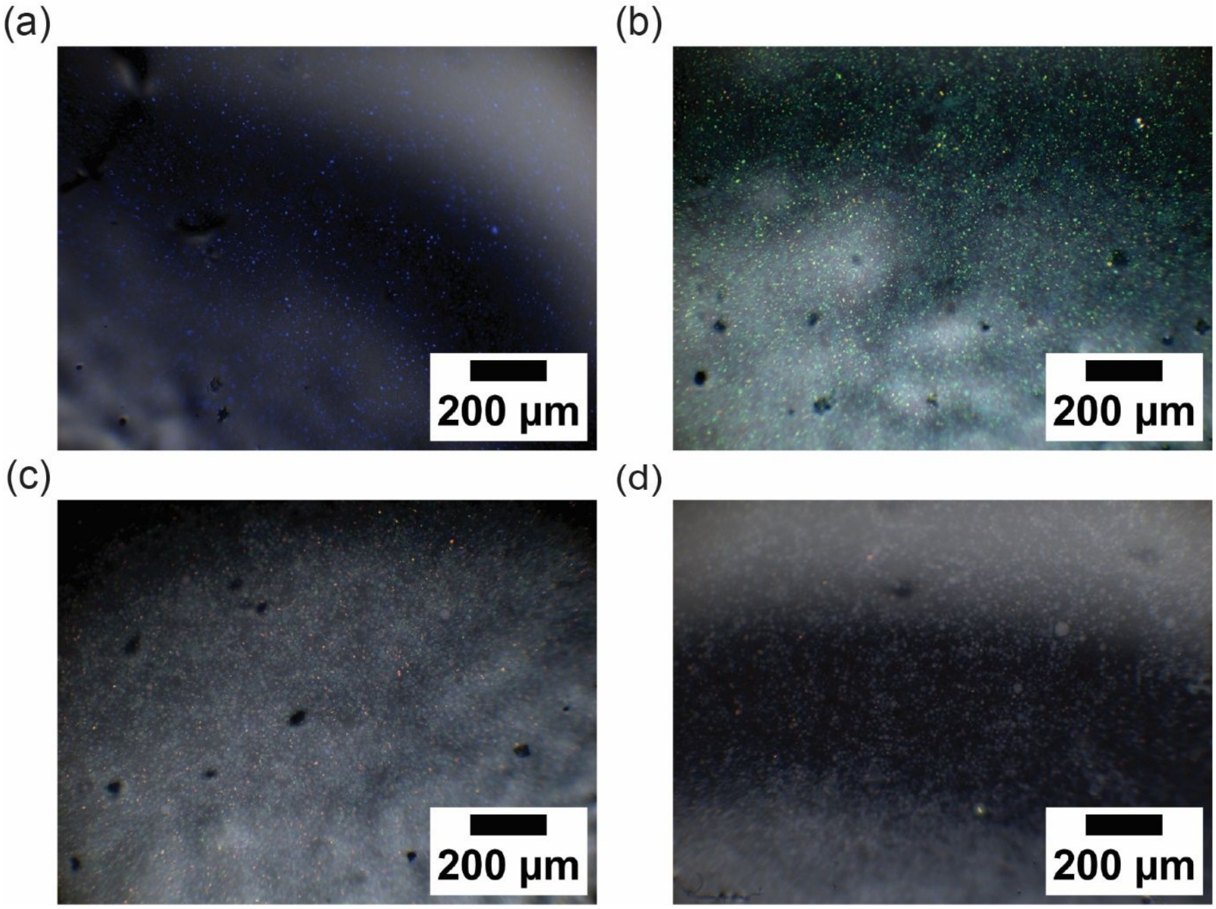

Figure S8. Optical microscopy images of photonic films obtained by drying optimized formulations (i.e., 1 wt% particle concentration and 15 wt% water-borne polyurethane concentration) of (a) P2VP(TIPh)-PMMA$(hPMMA)_{0.2}$ microparticles, (b) P2VP(TIPh)-PMMA$(hPMMA)_{0.4}$ microparticles, (c) P2VP(TIPh)-PMMA$(hPMMA)_{0.6}$ microparticles, and (d) P2VP(TIPh)-PMMA$(hPMMA)_{0.8}$ microparticles.

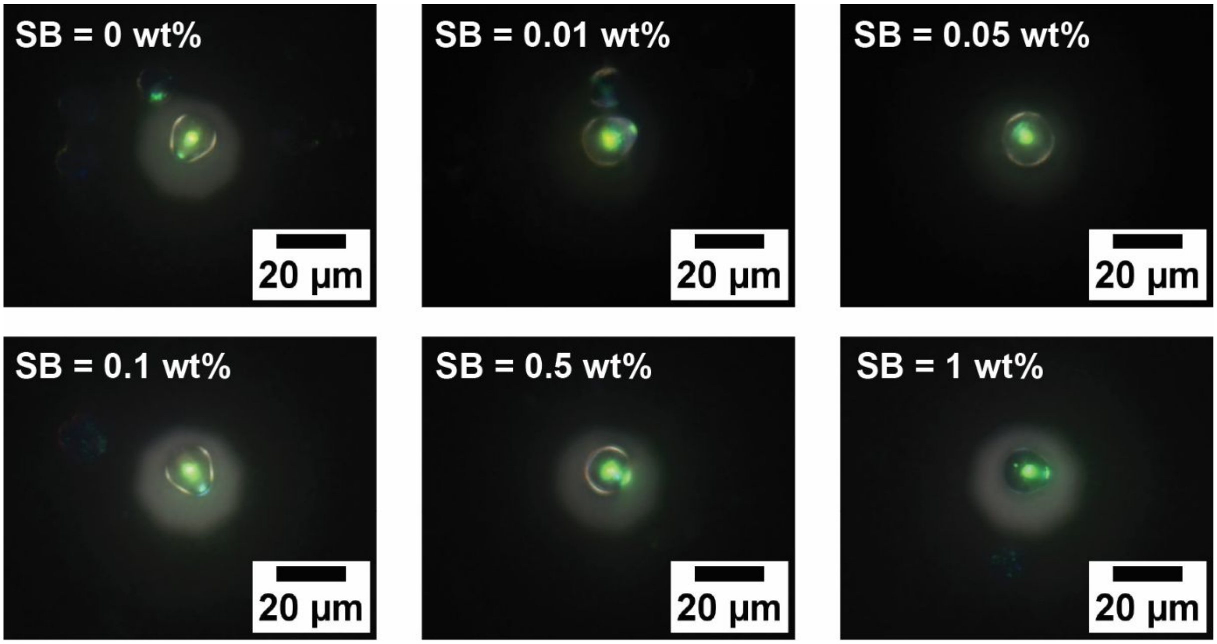

Figure S9. Optical microscopy images of P2VP(TIPh)-PMMA(hPMMA)$_{0.4}$ microparticles loaded with increasing amounts of SB, whose concentration is expressed with respect to the total amount of BCP and additives. A slight decrease in scattering effects is observed at higher SB concentrations.

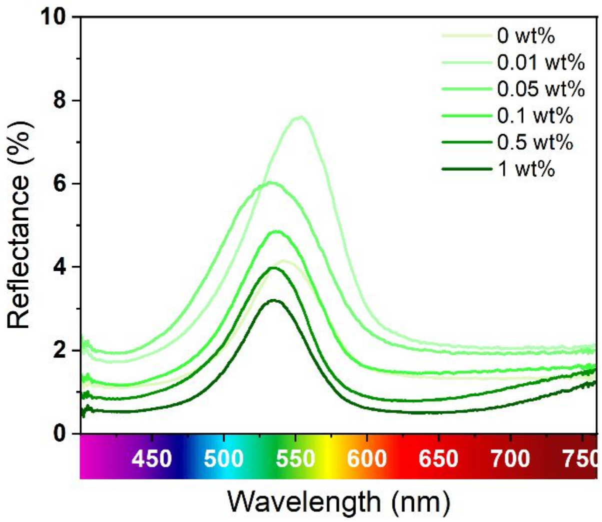

Figure S10. Reflectance spectra of P2VP(TIPh)-PMMA(hPMMA)$_{0.2}$ microparticles loaded with varying amounts of SB with respect to the total amount of BCP and additives.

Table S1. Detailed composition of the formulations used to prepare photonic microparticles with structural coloration spanning the entire visible spectrum. *x*-ratio is calculated as the ratio between TIPh molecules and 2VP repetition units, while hPMMA is added to counterbalance the volume increase associated with TIPh addition thus preventing the block copolymer symmetric nature.

x-ratio	BCP (mL)	TIPh (mL)	hPMMA (mL)
0	0.25	0	0
0.2	0.148	0.069	0.034
0.4	0.107	0.099	0.044
0.6	0.084	0.117	0.049
0.8	0.069	0.128	0.053
1	0.059	0.136	0.055

Table S2. Variables used to optimize the formulations of photonic paints.

Particle concentration (wt%)	WBPU concentration (wt%)
1	10
3	10
5	10
5	15
5	20
3	20
1	20
1	15
3	15
